# Use of Coffee Bean Bagasse Extracts in the Brewing of Craft Beers: Optimization and Antioxidant Capacity

**DOI:** 10.3390/molecules27227755

**Published:** 2022-11-10

**Authors:** Isabel H. Chacón-Figueroa, Luis G. Medrano-Ruiz, María de Jesús Moreno-Vásquez, Maribel Ovando-Martínez, Nohemí Gámez-Meza, Carmen L. Del-Toro-Sánchez, Daniela D. Castro-Enríquez, Guadalupe A. López-Ahumada, Ramón F. Dórame-Miranda

**Affiliations:** 1 Department of Chemical-Biological Sciences, University of Sonora, Hermosillo C.P. 83000, Sonora, Mexico; 2 Department of Scientific and Technological Research, University of Sonora, Hermosillo C.P. 83000, Sonora, Mexico; 3 Department of Food Research and Graduate Program, University of Sonora, Hermosillo C.P. 83000, Sonora, Mexico

**Keywords:** coffee bean bagasse, craft beer, bioactive compounds, functional beverage, additive, optimization

## Abstract

Coffee bean bagasse is one of the main by-products generated by industrial coffee production. This by-product is rich in bioactive compounds such as caffeine, caffeic and chlorogenic acid, and other phenols. The aims of this work are to optimize the extraction conditions of phenolic compounds present in coffee bean bagasse and incorporate them into stout-style craft beers, as well as to determine their effect on the phenol content and antioxidant capacity. The optimal conditions for extraction were 30% ethanol, 30 °C temperature, 17.5 mL of solvent per gram of dry sample, and 30 min of sonication time. These conditions presented a total phenol content of 115.42 ± 1.04 mg GAE/g dry weight (DW), in addition to an antioxidant capacity of 39.64 ± 2.65 μMol TE/g DW in DPPH^•^ and 55.51 ± 6.66 μMol TE/g DW for FRAP. Caffeine, caffeic and chlorogenic acids, and other minor compounds were quantified using HPLC-DAD. The coffee bean bagasse extracts were added to the stout craft beer and increased the concentration of phenolic compounds and antioxidant capacity of the beer. This work is the first report of the use of this by-product added to beers.

## 1. Introduction

Coffee is one of the world’s top beverages, obtained from the roasted and ground bean fruits of the coffee plant. Many environmental factors determine the coffee quality, as well as the aroma, flavor, and color; however, the climate and the location of the geographical area are some of the most important factors [1,2]. In Mexico, 944,413 tons of coffee are produced per year [2]. In 2019, coffee production reached more than 170 million bags (60 kg), mainly in South America. Brazil is the principal producer, followed by Colombia and Vietnam. Two main species of coffee are produced, *Coffea arabica* (69–74%) and *Coffea robusta* (23–30%). Additionally, coffee production generates large amounts of by-products such as coffee husks, pulp, skin, and bagasse [3]. The most significant amount of by-product (coffee bean bagasse) (45%) is generated during the preparation of coffee beverages [4]. The coffee bean bagasse is the waste generated from the coffee beverage manufacturing process; it represents 10% of the dry fruit; however, it represents 50% of the disposal after obtaining the coffee. Coffee bean bagasse is rich in protein, lignin, polysaccharides, minerals such as potassium, which is the most abundant, fats such as palmitic and linoleic acid, vitamin E, dietary fiber, and compounds formed by Maillard reactions such as melanoidins [5]. The caffeic and chlorogenic acids are the most important phenolic compounds in coffee bean bagasse since they play an essential role in health due to their antioxidant and sensory properties. Even though chlorogenic acid and its derivatives, methylxanthines, and caffeine (alkaloid), are the main compounds obtained during extraction; other phenolic compounds present are caffeoylquinic, feruloyl quinic, p-cumaroylquinic acids, mixed di-esters of ferulic, quinic and caffeic acid [6,7].

Caffeic and chlorogenic acid compounds are of great interest due to their potential applications in the food industry. The production of functional foods and beverages has increased due to their health benefits. For example, functional foods have been related to the prevention of cardiovascular, neurodegenerative, and tumor diseases [8]. This positive impact on human health is associated with the content or addition of bioactive compounds in food. Phenolic compounds, such as caffeic and chlorogenic acids, are among the main bioactive compounds used in functional foods and beverages [8]. They are biosynthesized from plants to act as biostimulants during plant growth. In addition, phenolic compounds can also have biological properties such as anti-inflammatory, anticancer, antiallergic, antiviral, and antioxidant [9]. An antioxidant compound, which inhibits, stops, or controls the oxidation of a substrate, can be defined as any substance present in low concentrations compared to an oxidizable substrate. This antioxidant compound can prevent or significantly delay substrate oxidation [10], a characteristic to consider as additive functional in beverages and improve the biological properties of functional foods.

Beer is one of the world’s oldest and most consumed alcoholic beverages, ranking third in popularity after coffee and water [11]. The base ingredients of any beer are malt, hops (*Humulus lupulus* L.), yeast, and water. Beer comprises prenylated flavonoids, phenolic acids, simple phenols, hydroxycoumarins, flavones, proanthocyanidins, tannins, and aminophenol compounds [12,13,14], which come mainly from malt and, to a lesser extent from hops, with a percentage of 70 and 30%, respectively [11,12]. Among those compounds, phenolic compounds can contribute to beer characteristics such as color, taste, haze, astringency, colloidal, and foam stability [15]. However, these compounds are reduced due to the filtration and clarification processes, low-quality raw materials, as well as storage conditions. Therefore, the addition of phenolic compounds can improve the health properties of manufactured beer [12]. Nowadays, the beverage industry seeks to add fruits and raw materials rich in bioactive compounds. The addition of fruits in the beer not only adds new flavors but also increases the content of bioactive substances with antioxidant capacity. Various fruits and adjuncts have been added to beer, such as omija, mango, persimmon fruit, raspberry, and cranberry juice. Additionally, the use of agro-industrial by-products, such as orange peels and eggplant peels, in the production of beer has also been explored [16]. Nevertheless, no work has been found regarding the addition of coffee bean bagasse to beer. Bioactive compounds in the coffee bean bagasse extracts could modify the physicochemical parameters, the polyphenols content, and the antioxidant activity in beers. Thus, the extracts of coffee bean bagasse could be an alternative to increase the profile and concentration of bioactive compounds in craft beer.

Therefore, this work aims to optimize the extraction conditions of phenolic compounds present in coffee bean bagasse and incorporate them into stout-style craft beers, as well as to determine their effect on the phenol content and antioxidant capacity.

## 2. Results

Table 1 shows the results of the experimental design matrix of phenols, flavonoid content, and antioxidant activity (DPPH^•^ and FRAP) in coffee bean bagasse under different extraction conditions. Four independent variables with three levels in this study were analyzed. The model presented 27 treatments where the concentration of the bioactive compounds was dependent on the sonication time (Table 1). The treatments that showed a higher concentration of total phenols were those extracted with ethanol at 30 % (*v*/*v*), 30 °C, 17.5 mL of solid/liquid ratio, and 30 min of sonication time. When using these parameters, a total phenols concentration of 115.42 ± 1.04 mg GAE/gDW, with an antioxidant capacity of 39.64 ± 2.65 μMol TE/g DW for DPPH^•^ assay and 55.51 ± 6.66 μMol TE/g DW for FRAP were obtained. However, the highest flavonoid content was not obtained following these conditions. Therefore, different extraction conditions, such as 60% ethanol concentration, 45 °C, 17.5 mL of solvent per gram of extract, and 30 min sonication time, were used to achieve the highest flavonoid content values. Under these conditions, a flavonoid content of 17.72 ± 0.04 mg QE/g DW, 28.10 ± 2.59 μMol TE/g DW for the DPPH^•^ assay, and 30.02 ± 4.20 μMol TE/gDW for FRAP were obtained.

### 2.1. Total Phenol and Flavonoid Content

The variance analysis of the phenolic optimization shows that the independent variables significantly affected temperature, sonication time, and the liquid-solid ratio (*p* ˂ 0.05). Furthermore, results showed significant differences in all the quadratic variables, in the interaction between ethanol concentration and sonication time, temperature and sonication time, and finally, the liquid-solid relationship and sonication time. Additionally, the coefficient of determination determined from variance analysis was R^2^ = 96.81%. This indicates that the model is adjusted to verify the behavior of the study. For the phenols content, a positive effect was observed in the liquid-solid relationship, sonication time, the quadratics of temperature, liquid-solid relationship, and sonication time, temperature, and liquid-solid relationship. Additionally, the interaction between the liquid-solid relationship and temperature, ethanol concentration and liquid-solid ratio, ethanol concentration, and sonication time were positively affected. On the other hand, ethanol concentration, temperature, the quadratics of ethanol concentration, the interactions of ethanol concentration and temperature, and temperature and sonication time, present a negative effect.

Regarding flavonoid content, it was noted that the independent variables were significant in temperature, sonication time, and the liquid-solid relationship (*p* ˂ 0.05). The quadratic variants that were found to be significant are ethanol concentration and temperature, the interaction concentration of ethanol and sonication time, temperature, and sonication time, and finally, liquid-solid ratio and sonication time, with a determination coefficient of R^2^ = 96.14%. According to this, the model was adjusted to verify the behavior of the study.

### 2.2. Effect of Independent Variables on the Total Extraction of Phenolic Compounds

#### 2.2.1. Effect of Ethanol Concentration

**Figure 1:**
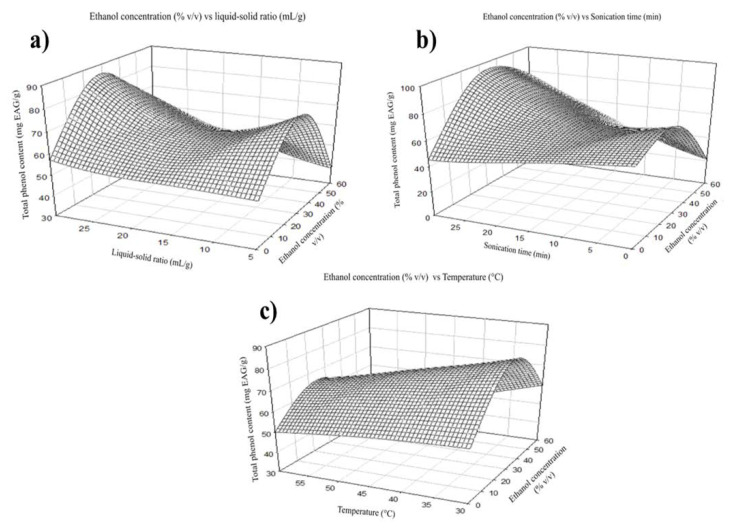
Effect of ethanol concentration on the extraction of total phenols (**a**) Graphical of ethanol concentration vs. liquid-solid ratio, (**b**) ethanol concentration vs. sonication time, and (**c**) ethanol concentration vs. temperature.

**Figure 2:**
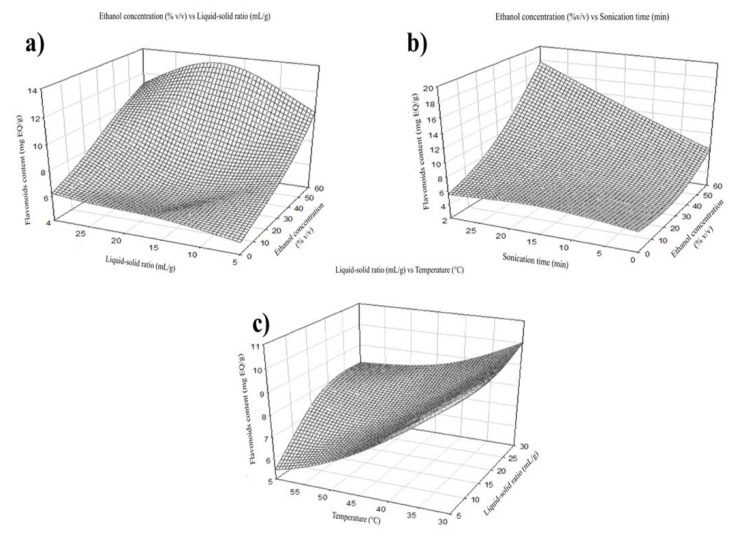
Effect of ethanol concentration on flavonoid extraction. (**a**) Graphical of ethanol concentration vs. liquid-solid ratio, (**b**) ethanol concentration vs. sonication time, and (**c**) liquid-solid ratio vs. temperature.

The extraction yield increase from using ethanol as a solvent since it is the most effective organic solvent to maximize the extraction of bioactive compounds [17]. In the case of total phenols, maximum extraction was obtained at a concentration of 30% ethanol (*v*/*v*) (Figure 1). Contrary to this, the recovery of flavonoids increases as the ethanol concentration increases, reaching its maximum extraction at a concentration of 60% ethanol (*v*/*v*) (Figure 2).

#### 2.2.2. Effect of Temperature, Liquid-Solid Ratio, and Sonication Time

**Figure 3:**
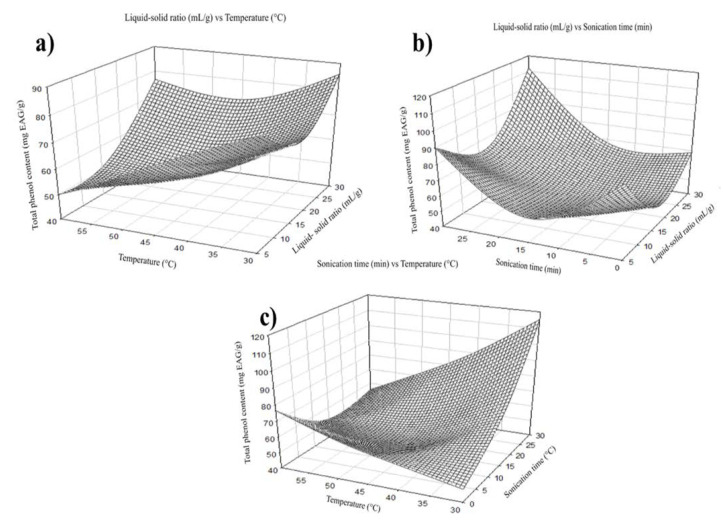
Effect of temperature, liquid-solid ratio, and sonication time on the extraction of total phenols. (**a**) Graphical of liquid-solid ratio vs. temperature, (**b**) liquid-solid ratio vs. sonication time and (**c**) sonication time vs. temperature.

**Figure 4:**
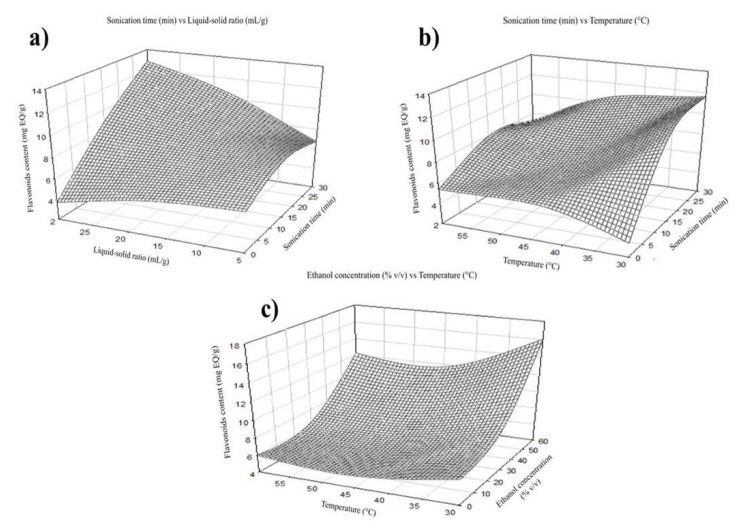
Effect of temperature, liquid-solid ratio, and sonication time on the extraction of flavonoids. (**a**) Graphical of sonication time vs. liquid-solid ratio, (**b**) sonication time vs. temperature, and (**c**) ethanol concentration vs. temperature.

The extraction performance was improved by increasing temperature as it enhances the solubility and reduces the solvent’s viscosity [17]. The total phenols were positively affected by the independent variable temperature. The maximum extraction was shown at 30 °C in the case of total phenols (Figure 3a). Still, flavonoid recovery reached its maximum extraction at 45 °C (Figure 4b). The maximum extraction yield of flavonoids and total phenols was 17.5 mL of solvent per gram of sample. This is maybe because a higher volume of solvent can extract more soluble phenolic compounds; however, the interaction with other factors may affect extraction (Figure 3a and Figure 4a) [18]. The highest recovery of flavonoid compounds and phenolic acids was 30 min in both cases.

### 2.3. Identification of Phenolic Compounds by HPLC-DAD

Table 2 shows the identification and quantification of phenolic compounds in the optimized coffee bean bagasse extract, craft beer, and craft beer produced with coffee bean bagasse extract. External standard compounds were used to identify and quantify the phenolic compounds in the extracts based on the retention time (Tr), maximum absorbance wavelength (λ max), and the elution profile of the metabolites. The presence of molecules such as gallic, caffeic, and chlorogenic acids, as well as the presence of caffeine, synaptic acid, p-coumaric acid, and syringic acid in the coffee bean bagasse, was detected. On the other hand, in the stout-style craft beer, the presence of molecules such as epicatechin, catechin, and epigallocatechin gallate was observed.

### 2.4. Brewing Craft Beer with Coffee Bean Bagasse Extract

**Figure 5:**
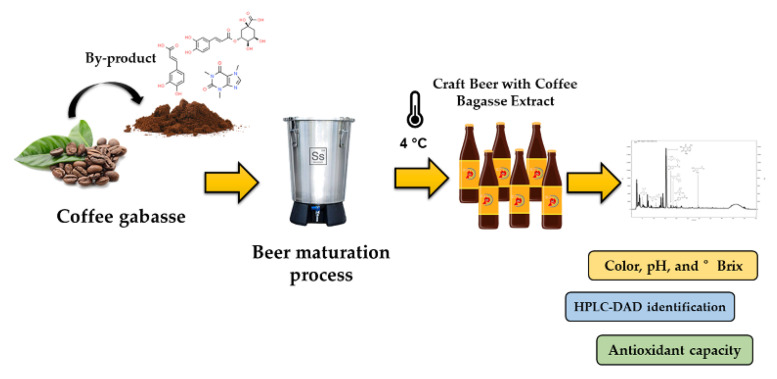
Schematic diagram of the brewing process with coffee bean bagasse extract.

Figure 5 shows a schematic diagram of the brewing process. The optimized coffee bean bagasse extract was added in the maturation process or second fermentation. The incorporation of the extract at this point has the target of increasing the phenolic compounds and antioxidant capacity. Accordingly, craft beer with coffee bean bagasse extract was characterized and analyzed by colorimetric assay to determine the content of bioactive compounds in the beverage.

#### pH and °Brix of Craft Beer

The fermentation process of craft beer began by activating the yeast in the must at 18 °C. The initial pH and °Brix were 5.04 ± 0.47 and 15 ± 3.46, respectively. Over time, the pH started to decrease along with °Brix because the yeast uses the free sugars in metabolic processes, generating volatile organic acids and esters, which indicates a correct fermentation process.

### 2.5. Color of Craft Beer with Coffee Bean Bagasse Extract

Table 3 depicts the color results of craft beer with three different concentrations of coffee bean bagasse extract (0, 1, 5, and 10 mg/L). The color value ranged from 41.00 ± 0.05 to 41.58 ± 0.02 °SMR. According to the Standard Reference Method (SMR) scale, the expected range for ale-type beers is 13–50 °SMR, while the stout style should be greater than 40 °SMR [19,20].

### 2.6. Content of Phenols, Flavonoids Totals, and Antioxidant Capacity of Craft Beer with Coffee Bean Bagasse Extract

Table 3 shows the phenols and flavonoid content present in the craft beer with coffee bean bagasse extract at 1, 5, and 10 mg/mL of craft beer. The concentration of 1 mg of dry extract per mL of craft beer (S1) most favors the phenols and flavonoids content, showing values of 537.30 ± 7.24 mg GAE/g DW and 263.81 ± 4.19 mg of QE/mL DW, respectively. The antioxidant activity results are consistent with the phenols content, and it was higher in the S1 sample by the DPPH^•^ and FRAP methods.

## 3. Discussion

Different studies have reported the concentration of bioactive compounds does depend on the extraction conditions and the food matrix. The content of total phenols and flavonoids obtained at different extraction conditions were higher than those reported by Torres-Valenzuela et al. [21], who reported phenols content values around 60.10 mg GAE/g of the dry sample in extracts from coffee cherry pulp using 52% (*v*/*v*) ethanol. Although these authors reported extraction with great potential, the yields were lower compared to other published works. Other by-products that have been evaluated are the coffee husks, which were obtained after roasting and assessed by the effect of variables such as solvent polarity, temperature, and extraction time. The results indicated the conditions influence of the extraction yields of bioactive compounds and antioxidant activity. Furthermore, differences in the content of phenolic compounds may be due to the process of each sample and, crop location.

For the optimization of coffee bean bagasse, the coefficient of determination (R^2^ = 0.96) indicated that only 3.19% of the total variations are not explained by the model. The adjusted coefficient of determination (R^2^_adj_) for a good statistical model should be close to R^2^. The value of the R^2^_adj_ (0.93) is relatively similar to the R^2^ (0.96) and confirms that the model is highly significant. According to this model, the linear terms of differences in all quadratic variables, in the interaction between ethanol concentration and sonication time, temperature and sonication time, and finally, the liquid-solid relationship and sonication time were significant (*p* < 0.05). Although the linear term of ethanol concentration and the interactions of ethanol and temperature, ethanol and liquid-solid ratio, and finally, temperature and liquid-solid ratio were not significant (*p* < 0.05), the sonication time was the variable that had the most significant impact on the extraction. This is attributed to that ultrasonic energy destroys the cell walls of the matrix, promoting solvent penetration into the sample and increasing the mass transfer [22,23].

In flavonoid content, the coefficient that presented a positive effect in linear terms was the ethanol concentration, liquid-solid ratio, and sonication time. Regarding the quadratic terms of liquid-solid ratio, sonication time, the interactions of ethanol concentration and liquid-solid ratio, ethanol concentration and sonication time, temperature and liquid-solid ratio, and temperature and sonication time, a positive effect was also observed. In contrast with the above, the linear term of temperature, the quadratic terms of ethanol concentration and temperature, and the interaction of ethanol concentration and temperature show a negative effect. The coefficient of determination (R^2^ = 0.96) indicates that the model does not explain 3.85 % of the total variations. Therefore, the adjusted coefficient of determination (R^2^_adj_) for a good statistical model should be close to the R^2^. The value of the R^2^_adj_ (0.91) is close to the R^2^ (0.96) and confirms that the model is highly significant.

The maximum extraction of total phenols was obtained with an ethanol concentration of 30 % (*v*/*v*). This may be due to the principle of mass transfer, which means a lower aqueous ratio of ethanol has a higher concentration gradient, better diffusion, and, therefore, better extraction yield [24]. Thus, results may be related to the polarity of the solvent and the polyphenols’ solubility in the solvent. This is attributed to the fact that heat weakens the interaction of phenolic compounds with proteins and polysaccharides, softening the cellular tissue; therefore, more compounds migrate to the solvent. However, caution must be exercised with the maximum temperature limit to avoid degrading the bioactive compounds [25]. The degradation temperature of bioactive compounds is above 80 °C. However, the degradation of bioactive compounds could be accelerated due to the presence of oxygen, metal ions, and UV radiation. 

The time of sonication and temperature is the principal independent variable that showed positive effects in the extraction. This may be because ultrasound favors the formation of tiny cavitation bubbles, which are subjected to compression and adiabatic expansion, which changes the temperature and pressure, breaking the cell wall. It has also been seen that ultrasonic movement can release bioactive compounds from the vacuole, so the higher the ultrasonic amplitude, the higher the extraction yield [26]. 

The phenolics compounds in the optimized coffee bean bagasse extract, craft beer, and craft beer produced with coffee bean bagasse extract were quantified and identified by HPLC. The craft beer produced with coffee bean bagasse extract showed a more diverse profile of phenolic compounds. The main phenolic compounds added to beer were caffeine, caffeic acid, chlorogenic acid, syringic acid, and sinapic acid. Previous studies have identified these molecules in green robusta coffee bean extract, where caffeine, chlorogenic acid, and caffeic acid are the main compounds. On the other hand, chlorogenic acid and caffeine were identified in 54 commercially available extracts of green coffee beans [27,28]. In addition, Pearson et al. [29] quantified chlorogenic and caffeic acid in dry raw herbs of Xu Duan (*Dipsacus asteroids*), showing retention times similar to those obtained in this work. On the other hand, Arai et al. [30] studied instant coffee and identified compounds such as trigonelline, caffeine, and chlorogenic acid. 

Studies carried out on ale-type craft beers have observed the presence of flavonol compounds such as procyanidins, catechin, and kaempferol 3-glucoside, with catechin being the most abundant [31]. The increase in ethanol concentration promotes the rise of phenolic compounds, which may be associated with their solubility. Furthermore, some yeasts can convert complex or glycosylated phenolic compounds into simple aglycones [32]. On the other hand, some compounds precipitate during maturation, which is associated with a decrease in bioactive compounds [33]. Furthermore, yeasts can degrade phenolic compounds as carbon substrates and promote their growth [34].

The significant decrease in pH and °Brix occurred in the first 48 h of fermentation, obtaining final pH and °Brix values of pH 4.01 ± 0.47 and 6.0 ± 3.46, respectively, resulting in a low alcohol concentration. However, this is not a significant factor in carrying out this work. The pH obtained is optimal since, according to reports by other authors, the pH range expected for ale-type beers is between 4–5. Therefore, the color of craft beers added with extract corresponds to the beer style. Beer color is provided by malt type and roast, adjuncts, hops, and fermentation pH [35]. In addition, the use of malts rich in bioactive compounds leached in the must and Maillard reactions during the boiling process [36]. 

The total antioxidant capacity of craft beer with and without coffee bean bagasse extract was determined by the FRAP method. It is possible to measure the ability to maintain the redox state in cells or tissues and the DPPH^•^ method in which the radical can be neutralized by direct reduction or electron transfer [37,38]. Table 3 shows the results of the antioxidant capacity of beer with the extract. The results of the antioxidant activity show that an antagonistic effect can occur by increasing the concentration of coffee bean bagasse extract in the beer (S5 and S10). However, comparing these results with the control beer, antioxidant values were higher in all treatments. This effect has already been reported in other studies. The compounds present in the extracts of coffee bean bagasse could act in a synergistic or antagonistic way. In this work, by increasing the extract concentration, the antioxidant activity decreases due to the interaction of the molecules with others [11,12]. This has an impact on the content of phenolic compounds and antioxidant activity. Therefore, the biological activity could be increased or not, depending on the type and structure of the molecules present. The concentration that showed the highest antioxidant activity was 1 mg of dry extract per mL of craft beer, obtaining 246.71 ± 13.89 μMol of TE/g DW in FRAP and 110.10 ± 2.44 μMol of TE/g DW for DPPH^•^ assay. The compounds present in the extraction of coffee bean bagasse added to craft beer showed a significant (*p* < 0.05) increase in the antioxidant activity of the final product. 

## 4. Materials and Methods

### 4.1. Samples

The 2-row base malt and caramel 60 were obtained from Malteurop. The coffee B and chocolate malts were obtained from The Swaen. Commercial oat flakes were acquired from a brewery supply store.The hops used were from the Cascade variety of American origin. The yeast used was *Saccharomyces cerevisiae* US-04 yeast from Fermentis. Coffee bean bagasse were donated by the coffee shop “Starbucks” in Hermosillo, Sonora.

### 4.2. Chemicals

Ethanol (Meyer, MX), methanol (Meyer, MX), 2,2-diphenyl-1-picrylhydrazyl (DPPH), 2,4,6-tris(2-pyridyl)-s-triazine (TPTZ), Folin-Ciocalteu (Sigma-Aldrich, EU), 6-hydroxy-2,5,7,8-tetramethylchroman-2-carboxylic acid (Trolox) (Sima-Aldrich, EU). All the other chemicals used were of analytical grade.

### 4.3. Drying and Storage of Coffee Bean Bagasse

The coffee bean bagasse was dried at 45 °C for 24 h in a convection oven (Heratherm, Thermo Scientific, Waltham, MA, USA) to reduce its moisture content. The coffee bean bagasse dried was stored without light in polyethylene bags at −20 °C for later analysis.

### 4.4. Optimization of Coffee Bean Bagasse Extraction Parameters

The extraction of phenolic and flavonoid compounds from coffee bean bagasse was done using a box-Behnken design. Table 4 shows the independent variables and their levels. First, 1 g of coffee bean bagasse was mixed with ethanol solutions following the conditions of the experimental design matrix and the 27 runs (Table 1). The suspensions were homogenized using a vortex (Vari-Whirl MixerVWR Scientific, San Francisco, CA, USA). Subsequently, the suspensions were ultrasonicated (Branson Sonicator, model 1510, Danbury, CT, USA) for 0, 15, or 30 min, then centrifuged at 5000× *g* (Heraeus Megafuge 16R Centrifuge, Thermo Scientific, Mexico City, Mexico) at 4 °C for 15 min. Finally, the supernatant was recovered, and the samples were filtered on the Whatman paper No. 2. The solvent was evaporated using a rotary evaporator (Labconco Digital rotary evaporator, Kansas City, MI, USA). The coffee bean bagasse extract obtained was frozen at −20 °C until further analysis.

### 4.5. Craft Beer Production with Coffee Bean Bagasse Extract

Stout craft beer was produced using 2-row base malt (2105 g) and a mixture of malts; caramel 60 (526 g), chocolate B (160 g), and black (120 g). In addition, cascade hops (25 g) were used at 25% of oat flakes, about the total weight of malt. The *Saccharomyces cerevisiae US-04* yeast was added for the fermentation process. After the beer fermentation process, the coffee bean bagasse extract was incorporated into the beer at different concentrations of control, 1 (S1), 5 (S5), and 10 (S10) mg/mL of craft beer (except for a control lot). 

#### Craft Beer Production Process

The production of the stout-style craft beer was based on what was described by Gasiński et al. [16], with some modifications. First, malts’ grain was ground in a roller mill with a separation of 0.5 mm between rollers (Pulvex 200). Later, the maceration was done using laboratory conditions under the following parameters: 10 L of initial purified water in 19 L stainless steel pots (SS Brewtech) and 0.21 g/L of calcium chloride were added to favor enzymatic activity. The malt mixture was mashed at a temperature of 65 °C for 60 min; this benefits the efficiency of starch hydrolysis and initiates the proteolytic action. Subsequently, they must be subjected to a recirculation process for 15 min to clarify and promote grain sedimentation. In addition, the residual grain was washed with 8 L of purified water at 70 °C. Finally, the resulting mixture (wort) was boiled for 60 min; at this stage, 25 g of hops of the cascade variety were added. The initial density was 15 °Brix, measured by a refractometer (Atago N1, 0.0–32.0% Brix ATC). During boiling, the Whirlpool technique was performed, and 1 g of Whirlfloc was added for a second clarification and sedimentation of proteins. The clarified beer was cooled with a plate cooler and placed in a fermenter for seven days (SS Brewtech) at a temperature of 18 °C. After this period, the beer was transferred to 500 mL glass fermentation flasks, to which coffee bean bagasse extract (except for control) was added. The concentrations used were 1 (S1), 5 (S5), and 10 (S10) mg of extract/mL of craft beer (except for the control). The beer maturation was held in a cold room at 4 °C for two days. Once the time had elapsed, the beer was bottled and subsequently analyzed. All treatments were performed in triplicate.

### 4.6. Color of Craft Beer and Craft Beer with Coffee Bean Bagasse Extract

The color of craft beer (control without extract), 1 (S1), 5 (S5), and 10 (S10) mg of extract/mL of craft beer was determined using the SRM method (Standard Reference Method). Determining the SRM value involved measuring the attenuation of light of a particular wavelength (430 nm) as it passes through 1 cm of beer, expressing the attenuation as absorption, and scaling the absorption by a constant. The absorbance (logarithm of light loss) is multiplied by 10, applying the Lambert-Bouger-Beer law [39].

SRM = 12.7 × A430

### 4.7. Flavonoid and Phenols Total Assay 

The total flavonoid content was determined by Kumaran and Karunakaran, [40]. A sample (80 µL of coffee bean bagasse extract, craft beer, and craft beer with coffee bean bagasse extract S1, S5, and S10). The extract was solubilized in an ethanolic solution of 30 % v/v, using a 10 mg/mL concentration. The craft beer and craft beer with coffee bean bagasse extract was diluted with distilled water in a 1:20 ratio for your analysis. The samples were added to 80 µL of AlCl_3_ at 20% using the ethanolic solution as solvent (96%) in a 96-well flat bottom microplate. After shaking for 30 s, the microplate was placed in the dark at 25 °C for 1 h. The absorbance of the reaction was measured at 415 nm on the plate reader. Quercetin hydrate was the standard and expressed total flavonoids as mg of quercetin equivalents (QE) per gram of dry weight (DW).

Total phenols were determined by the Folin-Ciocalteau method according to Singleton and Rossi, [41] with some modifications. First, 30 µL of samples in 150 µL of Folin-Ciocalteu 1N reagent (1:10) were mixed. Later, 120 µL of sodium carbonate (Na_2_CO_3_) at 7.5% was added. Next, it was left to stand for 30 min in the dark. Finally, the absorbance of the reaction was measured at 750 nm in a microplate reader (Multiskan FC, Thermo Scientific, Waltham, MA, USA). Gallic acid was used as a reference curve for all samples. Results were reported as mg gallic acid equivalents (GAE)/g DW.

### 4.8. DPPH^•^ Assay

The antioxidant capacity of the extracts was calculated by the method described by Palafox-Carlos et al. [42] with some modifications. First, 2.5 mg of the radical DPPH^•^ (2,2-diphenyl-1-picryl-hydracyl) was prepared in 80 mL of methanol; then, the solution was adjusted to an initial absorbance of 0.7 ± 0.05 before the reaction was carried out with 200 µL of radical and 20 µL of the samples (coffee bean bagasse extract, craft beer, and craft beer with coffee bean bagasse extract S1, S5, and S10). Then, the reaction was allowed to stand for 30 min in the dark and was read at a wavelength of 515 nm in a spectrophotometer equipped with a 96-well microplate reader (Multiskan FC, Thermo Scientific, Waltham, MA, USA). Then, the results were expressed as µM Trolox equivalents per gram of solution (µM TE/g DW), calculated based on a Trolox standard curve.

### 4.9. FRAP Assay

The FRAP assay was carried out using the Benzie and Strain, [43] method with some modifications. The FRAP reagent contains 2.5 mL of 10 μM TPTZ in 40 mM HCl plus 2.5 mL of 20 μM FeCl_3_ and 25 mL of 300 μM acetate buffer. After FRAP reagent preparation, 20 μL of the samples (coffee bean bagasse extract, craft beer, and craft beer with coffee bean bagasse extract S1, S5, and S10) were placed in each well of a microplate and mixed with 280 μL of FRAP solution. Samples were incubated at room temperature in the dark for 30 min, and absorbance was measured at 630 nm. The results were reported as µM Trolox equivalents per gram of dry weight (µM TE/g DW).

### 4.10. Identification and Quantification of Phenolic Compounds by HPLC-DAD

To begin the analysis, optimized coffee bean bagasse extract, craft beer (control), and craft beer with coffee bean bagasse extracts (S10) were filtered with a nylon filter (0.22 µm) and injected into the chromatographic system (50 µL). Afterward, the phenolic compounds were identified and quantified using an HPLC coupled to a diode array detector (HPLC-DAD, 1260 Infinity model, Agilent Technologies, Inc., Santa Clara, CA, USA). The separation of the compounds was done with a C-18 HPLC column (5 µm, 25 cm × 4 mm, Supelcosil^TM^ LC-18, SUPELCO). The solvent flow rate was 1.5 mL/min. The mobile phase A was acidified water with 5% formic acid, while the mobile phase B was methanol. Elution was done using a linear gradient from 100 to 98% A in 2 min, down to 68% A in 30 min, from 68–60% A in 8 min, from 60 to 5% A in 10 min, and 5% A in 5 min [44]. 

The chromatograms were recorded at 260, 280, 320, 330, and 360 nm. The identification of phenolic compounds was done by comparing the peak retention times and UV spectra of the samples against data obtained from commercial standards curves (Figure S1 in Supplementary materials). The compounds were quantified using standard curves of gallic acid, catechin, caffeine, caffeic acid, epigallocatechin gallate, chlorogenic acid, epicatechin, syringic acid, p-coumaric acid, and synapic acid (Sigma-Aldrich). Table S1 (Supplementary materials), shows the method validation parameters for phenolic compounds by HPLC-DAD (Analytical curve, limits of detection (LOD) and quantification (LOQ), and Linearity). The concentration of each compound present in the samples was expressed as µg per g of sample (µg/g) as the mean (*n* = 3) ± standard deviation.

### 4.11. Experimental Design and Statistical Analysis

The Box-Behnken design was used to evaluate the optimal conditions of the coffee bean bagasse extraction parameters. Statistical analysis was performed using a JMP 8 statistical package. The independent variables evaluated were ethanol concentration (% *v*/*v*), temperature (°C), liquid/solid ratio (mL/g), and sonication time (minutes), with three levels each (−1, 0, +1), data presented in Table 4. The response variable was the concentration of phenols (mg GAE/g DW) and content of flavonoids (mg QE/g DW), for which 27 experiments were performed in triplicate. A second-order polynomial mathematical equation expressed the relationship between the independent variables and the response, and the generalized form was as follows. Additionally, the general equation of the model is shown in Equation (1).

(1) $$y=\alpha_{0}+\Sigma \alpha_{i}x_{i}+\Sigma \alpha_{ii}x_{i}^{2}+\Sigma \alpha_{ij}x_{i}x_{j}+e$$

In the Equation (1), $\alpha_{i},\;\alpha_{ii},\;y\;\alpha_{ij}$ are coefficients of linear, quadratic, and interaction effects, respectively. Subsequently, 3D response surface plots were developed to study the interactive effect of the independent variables on the response. 

### 4.12. Statistical Analysis

The statistical analysis was performed using JMP software version 8 (SAS Institute, Cary, NC, USA). The validation of the developed model was carried out through an analysis of variance (ANOVA). Finally, the optimization was used to establish the process variables and thus obtain the highest concentration of phenols and flavonoids. The optimized coffee bean bagasse extract was used in the stout-style craft beer. An ANOVA and comparison of means with Tukey’s test (*p* < 0.05) were performed, as well as descriptive statistics.

## 5. Conclusions

In this study, the maximum extraction conditions of the phenolic compounds present in the coffee bean bagasse were optimized. The coffee bean bagasse extract presented a total phenol content of 115.42 ± 1.04 mg GAE/g DW, with to an antioxidant capacity of 39.64 ± 2.65 μMol TE/g DW in DPPH^•^ and 55.51 ± 6.66 μMol TE/g DW for FRAP using the optimal conditions of 30% ethanol, 30 °C temperature, 17.5 mL of solvent per gram of dry sample, and 30 min of sonication time. The main phenolics presented in the samples were chlorogenic acid, caffeic acid, and caffeine. After the addition of coffee bean bagasse extracts, the craft beer showed an increase in the content of the final product’s phenolics, flavonoids total, as well as antioxidant capacity. The coffee bean by-product extracts incorporated in craft beer could help to improve the health benefits.

## Figures and Tables

**Table 1:** Matrix of the experimental design with the response variables.

	Independent Variable				Response Variable			
Run	EtOH (% *v*/*v*)	Temperature (°C)	Solid/Liquid Ratio (mL/g)	Sonication Time (min)	Total Phenols Content (mg GAE/g DW)	Flavonoids Content (mg QE/g DW)	DPPH^•^ (μMol TE/g DW)	FRAP (μMol TE/g DW)
1	30	45	5.0	30	89.91 ± 1.11	6.76 ± 0.22	4.71 ± 2.28	29.83 ± 3.08
2	30	45	30.0	0	68.60 ± 3.76	4.58 ± 0.09	15.86 ± 0.64	15.38 ± 4.93
3	60	60	17.5	15	32.79 ± 0.02	12.25 ± 0.08	9.44 ± 1.79	20.35 ± 2.19
4	60	45	17.5	30	77.79 ± 0.28	17.72 ± 0.04	28.10 ± 2.59	30.02 ± 4.20
5	30	45	30.0	30	112.08 ± 2.81	12.88 ± 0.19	28.78 ± 2.48	26.94 ± 2.56
6	0	45	30.0	15	57.15 ± 2.73	6.23 ± 0.16	18.62 ± 0.99	16.06 ± 2.21
7	30	30	5.0	15	82.35 ± 1.02	9.64 ± 0.13	26.73 ± 2.84	29.85 ± 5.86
8	30	60	30.0	15	76.57 ± 0.24	8.11 ± 0.04	12.80 ± 2.61	31.01 ± 1.60
9	30	45	5.0	0	85.82 ± 2.11	5.82 ± 0.84	27.28 ± 1.80	27.37 ± 0.20
10	30	45	17.5	15	53.23 ± 1.41	5.32 ± 0.13	16.78 ± 1.96	16.05 ± 2.31
11	60	45	30.0	15	50.40 ± 0.33	11.16 ± 0.09	8.19 ± 1.32	17.74 ± 2.88
12	30	30	30.0	15	85.98 ± 3.01	10.02 ± 0.03	30.58 ± 4.47	53.16 ± 3.63
13	60	30	17.5	15	59.77 ± 2.80	16.05 ± 0.38	23.24 ± 1.83	35.41 ± 1.44
14	60	45	5.0	15	38.11 ± 2.36	10.39 ± 0.21	13.88 ± 1.87	15.55 ± 3.85
15	0	45	17.5	0	62.09 ± 2.65	4.89 ± 0.05	18.07 ± 1.30	28.79 ± 4.74
16	30	45	17.5	15	53.23 ± 1.41	5.32 ± 0.13	16.78 ± 1.96	16.05 ± 2.31
17	60	45	17.5	0	21.39 ± 0.96	7.62 ± 0.14	29.19 ± 0.75	29.43 ± 5.96
18	30	30	17.5	30	115.42 ± 1.04	11.64 ± 0.04	39.64 ± 2.65	55.51 ± 6.66
19	0	45	17.5	30	45.34 ± 1.08	5.49 ± 0.10	13.89 ± 2.16	13.21 ± 2.72
20	0	45	5.0	15	52.26 ± 0.34	4.94 ± 0.06	28.29 ± 0.30	20.46 ± 2.87
21	30	60	17.5	0	76.44 ± 1.78	5.40 ± 0.02	15.26 ± 0.01	17.62 ± 6.09
22	30	45	17.5	15	53.23 ± 1.41	5.32 ± 0.13	16.78 ± 1.96	16.05 ± 2.31
23	0	30	17.5	15	56.15 ± 0.66	6.80 ± 0.57	16.59 ± 1.18	25.00 ± 2.39
24	30	30	17.5	0	48.00 ± 0.62	3.32 ± 0.02	8.84 ± 0.92	12.35 ± 3.26
25	0	60	17.5	15	50.00 ± 1.79	5.97 ± 0.12	26.07 ± 0.74	21.02 ± 2.13
26	30	60	17.5	30	55.39 ± 1.64	7.16 ± 0.06	7.87 ± 1.08	15.87 ± 1.35
27	30	60	5.0	15	50.00 ± 3.89	5.45 ± 0.08	10.59± 1.02	11.89 ± 1.78

EtOH = Ethanol, GAE = Gallic acid equivalent, QE = Quercetin equivalent, TE = Trolox equivalent, DW = Dry weight.

**Table 2:** Identification and quantification of phenolic compounds content in coffee bean bagasse extracts, craft beer (control), and craft beer with coffee bean bagasse extract (S10) by HPLC-DAD (μg/g of sample).

	Samples				
Compounds	Retention Time (min)	Absorbance Wavelength (λ max in nm)	Optimized Coffee Bean Bagasse Extract	Craft Beer (Control)	Craft Beer with Coffee Bean Bagasse Extract (S10)
Gallic acid	5.0	280	477.17 ± 0.48	268.00 ± 4.49	153.98 ± 9.09
Catechin	10.8	280	ND *	77.71 ± 5.17	395.73 ± 5.65
Caffeine	15.6	280	5526.55 ± 35.86	ND *	12,528.31 ± 63.41
Caffeic acid	17.3	280	122.89 ± 2.68	ND *	130.58 ± 2.69
Epigallocatechin gallate	17.4	280	ND *	140.75 ± 4.16	731.73 ± 3.18
Chlorogenic acid	17.6	280	129.33 ± 4.92	ND *	227.27 ± 2.03
Epicatechin	18.7	280	ND *	213.26 ± 5.58	776.00 ± 0.02
Syringic acid	20.2	280	108.09 ± 0.86	ND *	77.88 ± 1.24
p-coumaric acid	20.4	280	261.11 ± 0.16	53.72 ± 0.18	114.64 ± 9.94
Sinapic acid	29.9	280	24.27 ± 0.07	ND *	30.48 ± 1.53

* Means with the same letters are not significantly different (*p* > 0.05). Values represent means (*n* = 3) ± standard deviation. ND: No Detected.

**Table 3:** Color (°SMR), concentration de phenols, flavonoids total, and activity antioxidant in craft beer with coffee bean bagasse extract.

Sample	Color (°SMR)	Total Phenol Content (mg of GAE/g DW)	Flavonoid Content (mg of QE/g DW)	DPPH^•^ (μMol of TE/g DW)	FRAP (μMol of TE/g DW)
**Control**	41.00 ± 0.05 ^a^	13.26 ± 0.94 ^a^	4.20 ± 0.48 ^a^	1.54 ± 0.14 ^a^	4.36 ± 0.49 ^a^
**S1**	41.41 ± 0.30 ^a^	537.30 ± 7.24 ^b^	263.81 ± 4.19 ^b^	110.10 ± 2.44 ^b^	246.71 ± 13.89 ^b^
**S5**	41.47 ± 0.14 ^a^	257.80 ± 3.36 ^c^	88.11 ± 1.54 ^c^	48.20 ± 2.02 ^c^	89.13 ± 0.51 ^c^
**S10**	41.58 ± 0.02 ^a^	115.01 ± 1.95 ^d^	52.16 ± 0.33 ^d^	33.31 ± 2.39 ^d^	55.36 ± 6.03 ^d^

The same letters mean significantly different (*p* > 0.05). Control = 0 mg/mL, S1 = 1 mg/mL, S5 = 5 mg/mL, S10 = 10 mg/mL. SMR = Standard Reference Method, GAE = Gallic acid equivalent, QE = Quercetin equivalent, TE = Trolox equivalent.

**Table 4:** Independent variables and levels were used in the optimization design.

	Factors	Levels		
Independent variables	X	−1	0	+1
Ethanol concentration (% *v*/*v*)	X_1_	0	30	60
Temperature (°C)	X_1_	30	45	60
Liquid/Solid ratio (mL/g)	X_2_	5.0	17.5	30
Sonication time (min)	X_3_	0	15	30

## Data Availability

Not applicable.

## Supplementary Materials

The following supporting information can be downloaded at: https://www.mdpi.com/article/10.3390/molecules27227755/s1, Figure S1: Graphical representation (*n* = 2 replicates) of the calibration curve of caffeine; Table S1: Method validation parameters for phenolic compounds by HPLC-DAD (Analytical curve, limits of detection (LOD) and quantification (LOQ), and Linearity).

## Sample Availability

Samples of the compounds are available from the authors.
