# Supplementary material for: Use of Coffee Bean Bagasse Extracts in the Brewing of Craft Beers: Optimization and Antioxidant Capacity
Source: Molecules. 2022 Nov 10;27(22):7755. doi: 10.3390/molecules27227755 (PMC9697320; doi:10.3390/molecules27227755)
Supplement: Supplementary file 1 [file molecules-27-07755-s001.zip › molecules-1931537-supplementary.pdf]

**Table S1.** Method validation parameters for phenolic compounds by HPLC-DAD (Analytical curve, limits of detection (LOD) and quantification (LOQ), and Linearity).

| Compounds[a] | Analytical curve | Absorbance wavelength (λ max in nm) | LOD (mg/mL) | LOQ (mg/mL) | Linearity ($R^2$) |
|---|---|---|---|---|---|
| Gallic acid | y = 175,657,807.728x - 762,067.437 | 280 | 0.018 | 0.055 | 0.982 |
| Catechin | y = 23,609,573.445x - 38,132.912 | 280 | 0.007 | 0.021 | 0.997 |
| Caffeine | y = 95,192,334.561x + 317,642.498 | 280 | 0.030 | 0.093 | 0.984 |
| Caffeic acid | y = 443,162,595.891x - 263,498.186 | 280 | 0.003 | 0.011 | 0.998 |
| Epigallocatechin gallate | y = 39,028,042.430x - 50,321.872 | 280 | 0.010 | 0.031 | 0.993 |
| Chlorogenic acid | y = 242,049,126.049x - 26,537.984 | 280 | 0.005 | 0.016 | 0.997 |
| Epicatechin | y = 26,264,718.017x - 18,525.645 | 280 | 0.010 | 0.031 | 0.993 |
| Syringic acid | y = 223,491,378.727x - 164,178.469 | 280 | 0.05 | 0.018 | 0.998 |
| p-coumaric acid | y = 469,296,076.475x - 1,060,244.839 | 280 | 0.014 | 0.043 | 0.986 |
| Sinapic acid | y = 422,664,972.800x - 44,328.913 | 280 | 0.020 | 0.061 | 0.997 |
| Myricetin | y = 323,163,573.044x + 67,209.101 | 360 | 0.003 | 0.011 | 0.999 |

[a]Concentration range 0-0.2 mg/mL

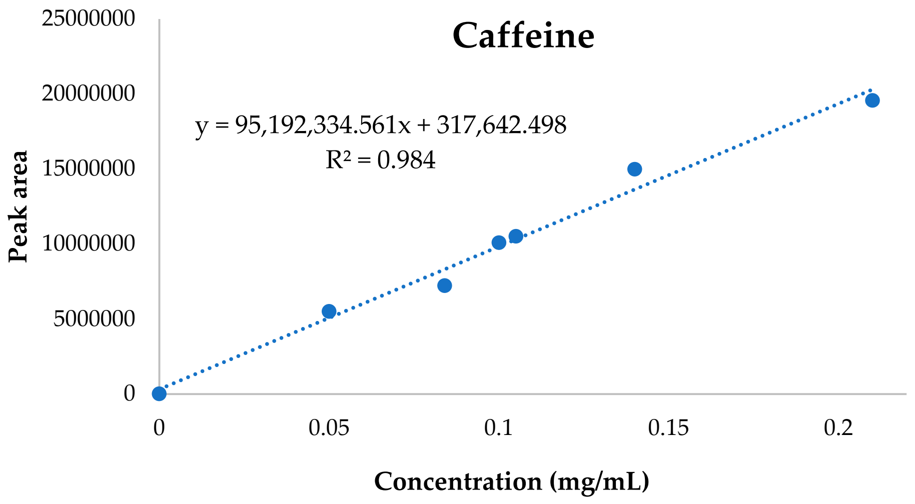


**Figure S1.** Graphical representation (n=2 replicates) of the calibration curve of caffeine.
